# Association between statin use and erectile dysfunction: Results from the National Health and Nutrition Examination Survey

**DOI:** 10.1097/MD.0000000000044423

**Published:** 2025-10-31

**Authors:** Sheng Chen, Jianqiang Zhang

**Affiliations:** a Department of Urology, The Ninth People’s Hospital of Nanning, Nanning, Guangxi, China; b Graduate School, Guangxi University of Chinese Medicine, Nanning, China; c Ruikang Hospital, Guangxi University of Chinese Medicine, Nanning, China.

**Keywords:** BMI, erectile dysfunction, NHANES, relationship, statin

## Abstract

This study aimed to examine the association between statin use and the prevalence of erectile dysfunction (ED) among a diverse cohort of American men aged 20 years and older, utilizing data from the National Health and Nutrition Examination Survey for the years 2001 to 2004. The analysis employed Logistic regression was used to assess the relationship between self-reported statin use and ED, adjusting for potential confounders, including age, ethnicity, educational level, smoking habits, diabetes mellitus, body mass index, alcohol consumption, hypertension, and hyperlipidemia. The initial participant pool consisted of 21,161 individuals, which was narrowed down to 3767 eligible subjects after applying the exclusion criteria. ED was evaluated based on the participants’ self-assessment of their ability to obtain and maintain an erection sufficient for satisfactory sexual performance. The study identified a statistically significant association between statin use and an increased prevalence of ED, with an odds ratio of 4.66 (95% confidence interval [CI]: 3.74–5.81) in the crude model. After adjustments for demographic, lifestyle, and health factors, the association remained significant, albeit reduced (odds ratio: 1.77, 95% CI: 1.34–2.35). Subgroup analysis highlighted variable correlation levels among different demographic and health-related groups, with age and body mass index identified as potential modifiers of the relationship between statin use and ED. Our findings indicate a positive correlation between statin use and ED prevalence in a representative sample of the American male population. This association, persistent across various subgroups and after adjusting for multiple confounding factors, underscores the need for further research to elucidate the underlying biological mechanisms and inform clinical decision making. The implications of this study suggest the necessity for a balanced approach in prescribing statin, particularly among populations at risk of ED, highlighting the importance of considering potential side effects in managing cardiovascular risk factors.

**Key points**

1. Comprehensive analysis of National Health and Nutrition Examination Survey data (2001–2004): This study provides an exhaustive analysis of the National Health and Nutrition Examination Survey data from 2001 to 2004, focusing on the association between statin use and erectile dysfunction (ED) among American men aged 20 years and older.
2. Significant association found: We discovered a statistically significant association between statin use and an increased prevalence of ED. Even after adjusting for potential confounders, statin users had higher odds of reporting ED compared to nonusers, underscoring a potential link between statin medication and sexual health issues.
3. Subgroup analysis for personalized insights: Subgroup analyses revealed that the association between statin use and ED varies across different demographic and health-related groups, with age and body mass index identified as significant modifiers. This suggests that individual patient characteristics can influence the risk of ED associated with statin use.
4. Implications for clinical decision-making: The findings highlight the need for clinicians to consider the potential side effects of statin therapy on sexual health. This is particularly important for populations at higher risk of ED, advocating for a balanced approach in prescribing statins.
5. Call for further research: Our study underscores the necessity for additional research to explore the underlying biological mechanisms. Understanding the causal pathways could lead to improved clinical guidelines that minimize the adverse effects of statin on sexual function while managing cardiovascular risk factors.
6. Conclusion: This study adds important evidence to the discussion on statin use and its association with erectile dysfunction, providing valuable insights for healthcare providers, patients, and researchers. The nuanced understanding of how statin therapy may impact sexual health underscores the importance of personalized medicine in cardiovascular disease management.

## 1. Introduction

Erectile dysfunction (ED), the problem of achieving or maintaining erection for sexual activity, is common among men and increases with age.^[1]^ Projections indicate that by 2025, the ED will affect an estimated 322 million men globally.^[2,3]^ The condition is intricately linked to cardiovascular diseases owing to overlapping risk factors, including diabetes mellitus (DM), metabolic syndrome, sedentary lifestyle, smoking, pelvic or spinal injuries or surgeries, obesity, hypertension, hyperlipidemia, and depression.^[4]^ This shared risk profile, particularly with coronary heart disease, has led to the increased use of statins among ED patients. It is also essential to reassure individuals who are candidates for statin therapy but do not suffer from ED, addressing concerns regarding the potential relationship between statin use and ED.^[5]^

The effects of statins on male sexual health have been debated. Hall et al found no link between statin use and ED in 1812 men,^[6]^ whereas Bruckert et al reported increased ED among statin users.^[7]^ These discrepancies may result from differences in the study methods and participant demographics.

Our main aim was to examine the association between statin use and ED using the National Health and Nutrition Examination Survey (NHANES) data, providing crucial insights for ED treatment.

## 2. Methods

### 2.1. Data source

The NHANES dataset of the Centers for Disease Control and Prevention was used as the data.^[8]^ The NHANES dataset employed in our study is publicly available and no special permission is required to access it. Detailed information and data can be accessed through the Centers for Disease Control and Prevention website at [https://www.cdc.gov/nchs/nhanes], facilitating transparency and enabling reproducibility of our research findings.

### 2.2. Study population

**Figure 1.**
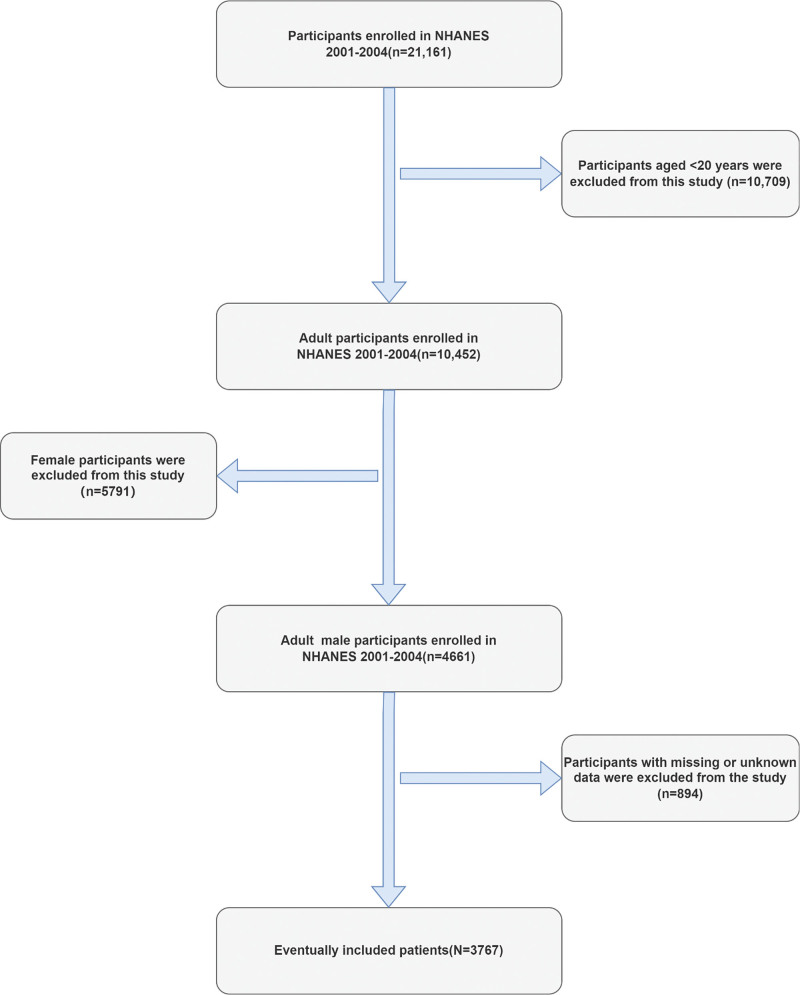
Flowchart of the study.

Between 2001 and 2004, data from surveys on “Medical Conditions” and “Prescription Medications” were meticulously categorized and collected. This research employed Logistic regression analysis was used to explore the link between ED and statin use, accounting for a variety of factors including age, ethnicity, educational level, smoking habits, DM, body mass index (BMI), alcohol intake, hypertension, and hyperlipidemia. Initially, the study included 21,161 participants. However, it was limited to individuals aged ≥20 years, which, after applying exclusion criteria, narrowed the participant pool to 3767. These participants provided information on various variables, including potential confounders, statin use, and instances of ED, which could influence the outcomes of the study (Fig. 1).

### 2.3. Assessment of statin use

The main independent variable of this study was self-reported statin use, as obtained from the NHANES, which records the use of prescribed medications within 30 days prior to completing the questionnaires. Participants were identified as undergoing statin therapy if they reported the use of any statin medication during this period. These individuals were included in the present study.

### 2.4. Assessment of ED

The study defined ED based on participants’ responses to the question, “How would you describe your ability to obtain and maintain an erection sufficient for satisfactory performance?” In this analysis, ED was defined as participants who answered “sometimes able” or “never able” to maintain an erection. Survey individuals who answered “always or almost always able” or “usually able” to keep an erection were classified as not having ED.

### 2.5. Covariates of interest

The study variables included age, race, education, smoking status, diabetes, BMI, alcohol consumption, high blood pressure, and cholesterol levels. The participants were categorized into 4 racial groups: Mexican American, Black, White, and Other. Educational levels were divided into “less than high school” for those who did not complete grade 12 and “high school or above” for those with at least a high school education.^[9]^ The NHANES defines hypertension as an average systolic or diastolic blood pressure of 140 or 90 mm Hg, respectively, based on 3 or 4 measurements.^[10]^ Heavy alcohol consumption (≥4 or ≥5 drinks/d) ≥5 d/mo, moderate alcohol consumption (≥3 drinks/d or ≥2 d/mo), or a history of daily binge drinking.^[11]^ Current alcohol use dummies were assigned as high, moderate, and light drinkers. Individuals who have never drunk (<12 drinks in life) or former drinkers (≥12 drinks in 1 year and no drinks in the past year).^[12]^ Poverty income ratio is household income divided by the poverty criterion of the survey year. Poverty income ratio is categorized as ≤1, 1–3, and ≥3.^[13]^ Doctors self-diagnosed DM or were treated with insulin or other medicines. Smoking status was determined using a cumulative cigarette intake of 100 cigarettes or more.^[14]^ Based on prior studies, we categorized the continuous variables for subgroup analysis. The following conditions indicate hyperlipidemia: male patients must have a total cholesterol, triglyceride, and low-density lipoprotein level of 150, 200, and 130 mg/dL, respectively, and be under 40 years old. Based on previous studies, we categorized the continuous variables for subgroup analysis. Two age groups were created: under 60 and over 60 years. BMI was categorized as normal (<25.0 kg/m^2^), overweight (25.0–29.9 kg/m^2^), or obese (≥30.0 kg/m^2^).^[15]^

### 2.6. Statistical analysis

Continuous data are presented as weighted mean values accompanied by their standard errors, while categorical data are expressed as numbers with corresponding weighted percentages. The selection of appropriate weights for analysis adhered to the guidelines outlined in the NHANES database instructions (available at https://www.cdc.gov/nchs/nhanes). Specifically, the Mobile Examination Center Exam Weight for 2-Year Data (WTMEC2YR) was utilized for analysis due to the inclusion of variables collected during Mobile Examination Center assessments. Furthermore, to account for the amalgamation of 2 NHANES survey cycles, the sample weight utilized in the final analysis was adjusted to half the value of “WTMEC2YR.”

Given the intricate sampling design of NHANES, all analyses conducted in this study incorporated sample weights, clustering, and stratification techniques to derive nationally representative estimates. Demographic characteristics related to ED status were assessed using chi-square and *t* tests.

The association between statin use and ED risk was examined through logistic regression analysis, which yielded odds ratio (OR) along with corresponding 95% confidence interval (CI). Adjustment procedures were implemented across several stages: initially, no adjustments were made in the crude model; subsequently, adjustments were applied for demographics in model 1; finally, adjustments were extended to encompass lifestyle and health factors in model 2. Stratified analyses were conducted based on age, education level, BMI, hypertension, and DM to assess the relationship between statin use and ED risk across different subgroups. All statistical analyses were performed using R, with statistical significance set at *P* < .05 to determine associations. This meticulous approach ensured robustness in the examination of the relationship between statin use and ED risk while maintaining methodological rigor throughout the study.

## 3. Results

### 3.1. Respondent baseline characteristics

The baseline characteristics are shown in Table 1. Participants with ED were more likely to be older, less educated, smokers, and have DM, hyperlipidemia, alcohol consumption, hypertension, and statin use.

**Table 1:** The baseline characteristics of the study participants.

Variable	Total	No ED	Have ED	*P* value
Statin use				<.0001
No	3264 (88.59)	2483 (92.40)	781 (72.28)	
Yes	503 (11.41)	223 (7.60)	280 (27.72)	
Age (yr)				<.0001
≥60	1192 (17.46)	436 (8.60)	756 (55.37)	
20–40	1341 (42.64)	1257 (50.15)	84 (10.49)	
41–60	1234 (39.90)	1013 (41.25)	221 (34.13)	
Ethnicity				.37
Mexican American	769 (7.77)	549 (7.99)	220 (6.85)	
Non-Hispanic Black	711 (9.77)	553 (10.09)	158 (8.39)	
Non-Hispanic White	2052 (74.29)	1425 (73.69)	627 (76.84)	
Other	235 (8.18)	179 (8.23)	56 (7.93)	
Poverty				<.0001
≤1	566 (10.52)	393 (10.48)	173 (10.70)	
≥3	1669 (56.32)	1297 (58.60)	372 (46.54)	
1–3	1532 (33.16)	1016 (30.92)	516 (42.76)	
Education				<.0001
>High school	1790 (56.31)	1372 (58.26)	418 (47.92)	
≤High school	1977 (43.69)	1334 (41.74)	643 (52.08)	
Diabetes mellitus				<.0001
Yes	485 (8.99)	193 (5.16)	292 (25.42)	
No	3282 (91.01)	2513 (94.84)	769 (74.58)	
Alcohol user				<.0001
Former	772 (16.62)	423 (13.45)	349 (30.18)	
Never	265 (6.89)	185 (6.88)	80 (6.90)	
Now	2730 (76.50)	2098 (79.67)	632 (62.92)	
Hypertension				<.0001
No	2244 (65.51)	1849 (71.11)	395 (41.52)	
Yes	1523 (34.49)	857 (28.89)	666 (58.48)	
BMI				.001
Normal weight	1128 (29.70)	843 (30.95)	285 (24.31)	
Obese	1090 (29.99)	758 (28.68)	332 (35.63)	
Overweight	1549 (40.31)	1105 (40.37)	444 (40.06)	
Smoke				<.0001
Former	1222 (29.06)	695 (25.02)	527 (46.36)	
Never	1519 (42.49)	1197 (45.17)	322 (30.99)	
Now	1026 (28.45)	814 (29.81)	212 (22.65)	
Hyperlipidemia				<.0001
No	1092 (29.36)	856 (31.41)	236 (20.57)	
Yes	2675 (70.64)	1850 (68.59)	825 (79.43)	

Statistical significance of differences in categorical variables was assessed using chisquare tests.

BMI = body mass index, ED = erectile dysfunction.

### 3.2. Association of statin utilization with ED

Table 2 shows the correlation between statin use and ED. Of the 3 logistic regression models tested, the use of statin was strongly associated with ED, with a high level of statistical significance (*P* < .001). The preliminary model indicated that patients who used statin had a 4.66 times higher likelihood of experiencing ED compared to individuals who did not use statin (OR: 4.66, 95%, CI: 3.74–5.81). We systematically adjusted for covariates from models 1 to 2 to minimize the influence of confounding factors. The correlation between statin use and the prevalence of ED progressively diminished, as other factors were considered. Ultimately, the utilization of statin continued to be substantially linked to a higher prevalence of ED (OR: 1.77, 95% CI: 1.34–2.35).

**Table 2:** Association between statin use and ED risks.

	Crude model		Model 1		Model 2	
Statin use	OR (95% CI)	*P*	OR (95% CI)	*P*	OR (95% CI)	*P*
No	Ref		Ref		Ref	
Yes	4.66 (3.74, 5.81)	<.0001	2.20 (1.71, 2.82)	<.0001	1.77 (1.34, 2.35)	<.001

Crude model: No adjustments for covariates.

Model 1: Adjustments for age, ethnicity (eth), education (edu), and poverty levels.

Model 2: Adjustments included ethnicity (eth), education (edu), age, poverty level, DM, smoking status, alcohol consumption, hypertension, BMI, and hyperlipidemia.

Logistic regression was used to calculate the OR and 95% CI for the association between statin use and ED, with *P*-values indicating statistical significance. “Ref” indicates the reference group, against which the OR for other groups is compared. In the context of statin use, “No” represents the reference group.

CI = confidence interval, ED = erectile dysfunction, OR = odds ratio.

### 3.3. Subgroup analysis

To evaluate the strength of the relationship between statin use and the occurrence of ED, subgroup analyses were conducted based on education, age, BMI, and the presence of hypertension. This analysis used the findings from Tables 1 and 2 as well as insights gained from clinical practice. All variables (covariates used for grouping were unadjusted) were corrected according to model 2. The results shown in Table 3 demonstrate various levels of correlation among the different demographic and health-related groupings. The data shown in Table 3 indicate that there are variable levels of correlation among various demographic and health-related subgroups in this study. Statins were found to have a favorable association with ED across all age groups. The *P*-value for the interaction term was .02, suggesting that the effect of statins on erectile function differed considerably depending on age. The impact of education level on the likelihood of experiencing ED while taking statins was significant. Individuals with high school education or lower had a substantially increased risk of developing ED with statins (OR: 3.20, 95% CI: 2.06–4.96, *P* < .0001). The risk was then divided based on body BMI categories, showing a substantially stronger correlation in obese persons (OR: 3.14, 95% CI: 1.81–5.45, *P* < .001). Our findings indicate that those without hypertension are more likely to experience ED related to statin use than those with hypertension are. The OR was 2.21, with a 95% CI: 1.24–3.95, and a *P*-value of .01. The OR for nondiabetic patients was 1.58 (95% CI: 1.14–2.17, *P* = .01), while for diabetic patients it was 2.11 (95% CI: 1.11–4.03, *P* = .03). The results of our study indicated that there were no statistically significant differences between the different groups (*P* for interaction >.05) in the impact of education, presence of hypertension, and presence of diabetes on the relationship between statin use and ED. Nevertheless, when examining the relationship between statin use and ED through stratified analyses, it was found that age and BMI may play a role in altering this correlation (*P* for interaction <.05).

**Table 3:** Subgroup analysis of statin use and ED association.

Subgroups	No statin use OR	Statin use OR (95% CI)	*P*	*P* for interaction
Age				.02
20–40	Ref	11.23 (1.75, 71.93)	.01	
41–60	Ref	2.50 (1.66, 3.76)	<.001	
≥60	Ref	1.41 (1.05, 1.89)	.03	
Education				.17
>High school	Ref	1.41 (0.88, 2.26)	.15	
≤High school	Ref	3.20 (2.06, 4.96)	<.0001	
BMI				.02
Obese	Ref	3.14 (1.81, 5.45)	<.001	
Normal	Ref	1.37 (0.59, 3.19)	.44	
Weight				
Overweight	Ref	1.71 (1.16, 2.50)	.01	
Hypertension				.8
No	Ref	2.21 (1.24, 3.95)	.01	
Yes	Ref	1.94 (1.33, 2.83)	.002	
DM				.09
No	Ref	1.58 (1.14, 2.17)	.01	
DM	Ref	2.11 (1.11, 4.03)	.03	

All data were adjusted for ethnicity, PIR, educational level, smoke, DM, alcohol, and hyperlipidemia (except for grouping covariates).

BMI = body mass index, CI = confidence interval, DM = diabetes mellitus, ED = erectile dysfunction, OR = odds ratio, PIR = poverty income ratio.

## 4. Discussion

Statins, which inhibit 3-hydroxy-3-methylglutaryl-coenzyme A reductase, control cholesterol by blocking the synthesis of mevalonate, which is essential for cholesterol production. This leads to reduced cholesterol and low-density lipoprotein cholesterol levels, with variations across statin types.^[16]^ Statins can cause side effects, including neurological, liver, and kidney issues, and cataracts.^[17,18]^ Studies have linked statin use to ED, including a clinical trial showing worsened erectile function in men after statin initiation^[19]^ and research suggesting a potential association between statin use and ED.^[20]^ One study found decreased libido and testosterone levels in men treated with various statin.^[21]^ Testosterone is crucial for male sexual function, affecting libido and erectile quality.^[22]^ Research indicates lower testosterone levels in men with sexual dysfunction,^[23]^ and another study found a higher incidence of ED among statin users, suggesting statins as a risk factor for ED.^[7]^ Statins may affect male sexual health by altering cholesterol availability for testosterone synthesis, which is crucial for the Leydig cells in the testes. Although statin reduce low-density lipoprotein-cholesterol, they may not impair steroidogenesis directly; however, they could affect cholesterol bioavailability for testosterone production.^[21,22,24]^ Subsequently, the lack of or decrease in testosterone will lead to ED.

Our study analyzed data from 3,767 NHANES participants between 2001 and 2004, finding a positive link between statin use and ED in a representative US sample.

After adjusting for various factors, our analysis identified a significant association between statin use and a higher prevalence of ED, OR of 1.77 (95% CI: 1.34–2.35). This association was consistently observed across different subgroups, indicating a notable relationship between statin use and an increased prevalence of ED.

Moreover, this finding highlights the need for additional research to understand the biological mechanisms underlying the association between statin use and erectile dysfunction. Such an understanding could pave the way for the development of treatment strategies that effectively manage cholesterol levels while minimizing their potential impacts on sexual function. Unraveling these mechanisms may assist in creating new therapeutic approaches that offer a balanced reduction in cholesterol levels, without compromising sexual health. Given the aging population and increasing risk of cardiovascular diseases, the use of statins is expected to increase. Therefore, elucidating the potential side effects of these medications is crucial to global public health. Future studies should aim to assess the relationship between statin use and erectile dysfunction and explore possible biological explanations to guide clinical decision-making and patient management, ultimately improving the quality of life of patients.

As we underscore the necessity for further research into the biological mechanisms linking statin use to erectile dysfunction, we must also critically reflect on the limitations inherent in our study. While our research, leveraging the NHANES’s diverse and representative sample, confirms the association between statin use and ED, its cross-sectional design precludes us from establishing causal relationships. This methodological constraint is crucial for interpreting our findings as it limits the extent to which we can infer the directionality of the observed association. Moreover, the reliance on self-reported data for both statin use and ED diagnosis introduces the potential for recall bias, which could affect the accuracy of the reported associations. The absence of precise data on the timing of statin initiation relative to the onset of ED symptoms further complicates our ability to discern a clear temporal relationship, necessitating a cautious approach to interpreting our results. Acknowledging these limitations is vital in setting the context within which our findings should be considered. They highlighted the need for longitudinal studies that can more definitively ascertain causal links and examine the impact of variables such as the dosage and duration of statin use on erectile function. Such research would significantly advance our understanding of the complex interplay between lipid management and sexual health, contributing to the development of treatment strategies that optimize cardiovascular outcomes without compromising sexual wellbeing.

## 5. Conclusions

In summary, this study identified a positive correlation between the use of statin medications and the prevalence of ED in a diverse cohort of American individuals aged 20 years. This finding suggests a potential link between statin medication patterns and sexual health issues across a broad demographic, underscoring the importance of considering medication’s side effects in clinical practice. Although this study provides significant initial evidence, further verification through prospective cohort studies is necessary. If future research confirms this association, it could have profound implications for public health policies and clinical practices, potentially leading to more cautious prescriptions of statins, especially among patients who may already be at risk of erectile dysfunction.

## Author contributions

**Conceptualization:** Sheng Chen.

**Data curation:** Sheng Chen, Jianqiang Zhang.

**Formal analysis:** Sheng Chen, Jianqiang Zhang.

**Funding acquisition:** Sheng Chen, Jianqiang Zhang.

**Investigation:** Sheng Chen, Jianqiang Zhang.

**Methodology:** Sheng Chen, Jianqiang Zhang.

**Project administration:** Sheng Chen, Jianqiang Zhang.

**Resources:** Sheng Chen, Jianqiang Zhang.

**Software:** Sheng Chen, Jianqiang Zhang.

**Supervision:** Sheng Chen.

**Validation:** Sheng Chen, Jianqiang Zhang.

**Visualization:** Sheng Chen, Jianqiang Zhang.

**Writing – original draft:** Sheng Chen, Jianqiang Zhang.

**Writing – review & editing:** Jianqiang Zhang.
